# GIDDY UP! LESSONS LEARNED FROM INTRODUCING AN ONLINE, NATURE-BASED INTERVENTION TO ASSISTED LIVING

**DOI:** 10.1093/geroni/igac059.3127

**Published:** 2022-12-20

**Authors:** Megan Westmore, Keith Anderson, Rebecca Mauldin

**Affiliations:** University of Texas at Arlington, Arlington, Texas, United States; University of Mississippi, Oxford, Mississippi, United States; University of Texas at Arlington, Arlington, Texas, United States

## Abstract

The processes involved with developing, funding, and conducting intervention studies in long-term care present a unique set of challenges for researchers. In this presentation, we explore the experiences of researchers evaluating an online nature-based intervention in assisted living. The first challenge lies in creating or finding a novel intervention specific to the setting and population. In this case, the researchers found and tailored an existing online, interactive program that focused on ranch life in rural Montana – Days at Dunrovin. In partnership with the developer of Days at Dunrovin, a group intervention was developed specifically for long-term care settings. The second challenge is finding a facility that has the desire, environment, and staffing to support the intervention. In this case, the researchers were able to partner with an assisted living facility that had an owner who was interested in research, an environment that matched the experimental design of the study, and a staff that was eager to find new ways for residents to engage with the world, particularly during the COVID-19 pandemic. The final challenge involves selecting a funding mechanism that best fits the needs of the study. In this case, the researchers targeted a private foundation that provided ample funding along with the latitude and flexibility that allowed the study to evolve and pivot as necessary. This presentation concludes with recommendations for both budding and seasoned researchers who seek to introduce creative interventions into long-term care settings.

